# Cost-effectiveness of nurse-led self-help for recurrent depression in the primary care setting: design of a pragmatic randomised controlled trial

**DOI:** 10.1186/1471-244X-12-59

**Published:** 2012-06-07

**Authors:** Karolien EM Biesheuvel-Leliefeld, Sandra MA Kersten, Henriette E van der Horst, Anneke van Schaik, Claudi LH Bockting, Judith E Bosmans, Filip Smit, Harm WJ van Marwijk

**Affiliations:** 1 Department of General Practice and the EMGO + Institute for Health and Care Research (EMGO+), VU University medical Centre, Amsterdam, The Netherlands; 2 Department of Psychiatry and the EMGO + Institute for Health and Care Research (EMGO+), VU University Medical Centre, Amsterdam, The Netherlands; 3 Department of Clinical Psychology, Groningen University, Groningen, The Netherlands; 4 Department of Health Sciences and EMGO + Institute for Health and Care Research, Faculty of Earth and Life Sciences, VU University Amsterdam, Amsterdam, The Netherlands; 5 Department of Public Mental Health, Trimbos Institute (Netherlands Institute of Mental Health and Addiction), Utrecht, The Netherlands; 6 Department of Epidemiology and Biostatistics, EMGO + Institute for Health and Care Research, VU University Medical Centre, Amsterdam, The Netherlands

**Keywords:** Major depressive disorder, relapse, recurrence, prevention, cognitive therapy, primary care, randomised controlled trial, design

## Abstract

**Background:**

Major Depressive Disorder is a leading cause of disability, tends to run a recurrent course and is associated with substantial economic costs due to increased healthcare utilization and productivity losses. Interventions aimed at the prevention of recurrences may reduce patients' suffering and costs. Besides antidepressants, several psychological treatments such as preventive cognitive therapy (PCT) are effective in the prevention of recurrences of depression. Yet, many patients find long-term use of antidepressants unattractive, do not want to engage in therapy sessions and in the primary care setting psychologists are often not available. Therefore, it is important to study whether PCT can be used in a nurse-led self-help format in primary care. This study sets out to test the hypothesis that usual care plus nurse-led self-help for recurrent depression in primary care is feasible, acceptable and cost-effective compared to usual care only.

**Design:**

Patients are randomly assigned to ‘nurse-led self-help treatment plus usual care’ (134 participants) or ‘usual care’ (134 participants). Randomisation is stratified according to the number of previous episodes (2 or 3 previous episodes versus 4 or more). The primary clinical outcome is the cumulative recurrence rate of depression meeting DSM-IV criteria as assessed by the Structured-Clinical-Interview-for-DSM-IV- disorders at one year after completion of the intervention. Secondary clinical outcomes are quality of life, severity of depressive symptoms, co-morbid psychopathology and self-efficacy. As putative effect-moderators, demographic characteristics, number of previous episodes, type of treatment during previous episodes, age of onset, self-efficacy and symptoms of pain and fatigue are assessed. Cumulative recurrence rate ratios are obtained under a Poisson regression model. Number-needed-to-be-treated is calculated as the inverse of the risk-difference. The economic evaluation is conducted from a societal perspective, both as a cost-effectiveness analysis (costs per depression free survival year) and as a cost-utility analysis (costs per quality adjusted life-year).

**Discussion:**

The purpose of this paper is to outline the rationale and design of a nurse-led, cognitive therapy based self-help aimed at preventing recurrence of depression in a primary care setting. Only few studies have focused on psychological self-help interventions aimed at the prevention of recurrences in primary care patients.

**Trial registration:**

NTR3001 (http://www.trialregister.nl)

## Background

### Recurrent major depressive disorder

Major Depressive Disorder (MDD) is a leading cause of disease burden and is associated with significant healthcare costs and costs stemming from productivity losses [1,2]. MDD’s disease burden stems largely from its recurrent nature [3]. Each additional depressive episode increases the risk of recurrence by 18 % [4]. Interventions aimed at the prevention of recurrences in recovered patients may significantly reduce the burden of depression [3]. Up till now, maintenance treatment has been largely based on antidepressants (AD). However, the evidence-base to support such prolonged treatment is poor [5-8] and moreover there is no evidence when to stop AD since most studies restricted their follow up to no longer than 2 years [9]. Furthermore, many depressed patients prefer psychological treatments to drugs [10] and many are not willing to take AD for prolonged periods of time [11,12]. Also, adherence in AD users is estimated at only 50 % at best [11-13]. As such, there is a need for an accessible alternative to maintenance treatment with AD. Psychological interventions might offer an interesting alternative to prevent recurrence of MDD in recovered patients.

### Terminology

**Figure 1:**
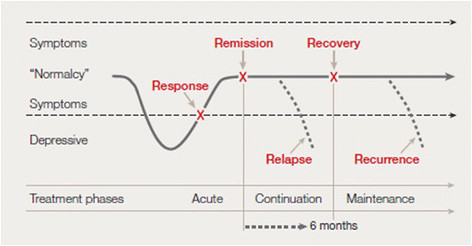
**Overview of response, remission, recurrence, and relapse in relation to the treatment phase**[14]**.** Modified after Tohen et al. (2009) [16] © 2009 Blackwell Munksgaard

At this point it might be well to introduce some terminology. MDD tends to run a relapsing and recurrent course. Both relapse and recurrence refer to the reappearance of a full-blown MDD after a symptom-free period. The essential distinction between both terms is the time at which each event occurs (Figure 1[14]). According to the description by Frank et al. [15], relapse is defined as ‘a return of symptoms satisfying full syndrome criteria for an episode that occurs during a period of remission, but before recovery’. As for recurrence, this is defined as ‘the appearance of a new episode of MDD, occurring during recovery’. Conceptually, this represents the beginning of a new episode of an illness. Treatment stages can be defined accordingly: following remission and before recovery ‘continuation treatment’ is offered. Following recovery, treatment enters the maintenance stage. Both treatments have the aim to prevent recurrences.

For research purposes a consensus definition recommends that a 6-month threshold is justifiable to distinct between remission and recovery because of the median duration of MDD [15]. In patients experiencing relapsing/recurring MDD however, this threshold can be debated because of the mostly shorter duration of an episode. In this trial no distinction is made between relapse and recurrence (henceforth called ‘recurrence’) nor between remission and recovery (henceforth ‘recovery’), nor between continuation and maintenance treatment (henceforth ‘maintenance treatment’). Exact definitions and cut-off points of all terms with respect to this trial are handled in detail later (see ‘Eligibility of participants’).

### Efficacy of psychological interventions

The literature shows that psychological interventions may offer a good alternative or a welcome adjunct to AD. Hollon et al. (2010) concluded in their review that Cognitive Behavioural Therapy (and especially CT) is as efficacious as medications in the treatment of MDD and that CT has an enduring effect that protects against subsequent relapse and possibly recurrence regardless of when it is applied [17]. Continuation/maintenance CT has been found to reduce risk for relapse/recurrence in MDD in a trial of Jarrett et al. (2001) [18]. A meta-analysis by Vittengl et al. (2007), including 28 studies and comprising 1,880 adults, demonstrated that among acute-phase treatment responders, continuation of CBT compared to assessment only or clinical management, reduced the number of recurrences substantially from 73 % to 40 %, over a mean of 153 weeks follow-up [19]. Specific protocols based on CBT have been developed for the prevention of depression, such as Preventive Cognitive Therapy (PCT). For patients with a history of 4 or more depressive episodes, 75 % receiving PCT experienced a recurrence over a period of 5,5 years versus 95 % receiving usual care [20]. PCT targets underlying cognitive vulnerability factors, such as dysfunctional cognitions that are easily reactivated in recovered patients and therefore may cause vulnerability for recurrence. The treatment focuses on identifying and changing these vulnerability factors, while at the same time reinforcing specific memories of positive experience by keeping a diary of positive experiences and formulating specific recurrence prevention strategies [21].

### The case for guided self-help

In the Netherlands, evidence-based psychological treatments are less readily accessible in primary care [10] because they require specific expertise, extensive training and draw on scarce resources. Furthermore, reimbursement of psychological treatments in primary care through insurance is becoming limited. Because the vast majority of persons with a high risk of developing a new episode visits - and receives treatment from - their own primary care physician (PCP), this seems to be the most appropriate coordinator of preventive interventions [22]. These interventions should be cost-effective, readily accessible at the primary care or community level, acceptable for patients and health care providers, and should easily be integrated into current care.

Self-help interventions using self-help books (bibliotherapy) are one of the most accessible forms of psychological interventions for primary care patients. Research indicates that self-help has a moderate to large effect in reducing symptoms of depression and anxiety [23-26]. PCT can easily be transformed into a self-help intervention because of its structured design. Some form of support however, should be provided to enhance patients' compliance, which in turn is associated with better treatment response overall [26-29]. There is growing evidence that mental health nurses or social workers can effectively deliver self-help treatment protocols for depression, particularly in chronic care models [30,31]. For both economic and pragmatic reasons it is attractive to let nurses play a pivotal and facilitating role in the provision of PCT instead of a psychologist.

### Objectives

The primary objective of this study is to evaluate whether nurse-led, cognitive treatment based self-help in addition to usual care is cost-effective in preventing recurrences for patients at high risk of recurrent MDD in primary care compared to usual care alone. Furthermore, this study examines whether the addition of nurse-led self-help to usual care for patients with recurrent MDD is effective in improving health related quality of life, in reducing co-morbid distress, anxiety and/or somatisation, in improving self-efficacy and meets with patients’ satisfaction. Finally, we examine which socio-demographic and clinical variables (e.g. pain and fatigue) moderate treatment response.

## Design

**Figure 2:**
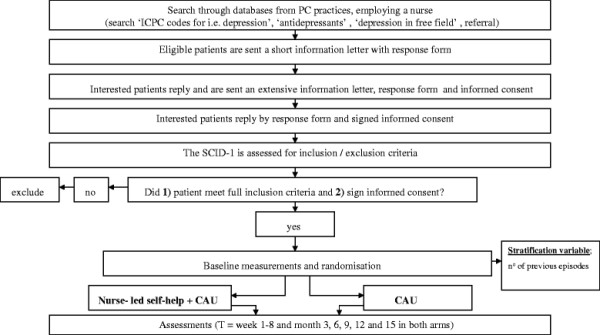
Flowchart.

This study is a multi-site, pragmatic randomised controlled trial among primary care patients with recurrent MDD who are currently recovered. Patients are recruited through primary care practices and are randomly assigned to two parallel groups: ‘nurse-led self-help treatment plus usual care’ (134 patients) or ‘usual care alone’ (134 patients). A flowchart is shown in Figure 2. It is not possible to blind neither patients nor healthcare providers to the intervention in this study due to the nature of this self-help intervention.

### Eligibility of participants

Patients are eligible for participation in the trial when they: 1) are between 18 and 65 years old, 2) have had at least 2 previous depressive episodes 3) are currently recovered, 4) are fluent in reading and speaking Dutch and 5) have access to the internet. Criterion for ‘currently recovered’ includes ‘no diagnose of depression according to the Structured Clinical Interview for DSM-IV (SCID-I)’ [32]. The recovered episode should last for longer than 8 weeks and no longer than 2 years.

Participants who have current (hypo) mania or a history of bipolar disease, any current organic brain disorder, psychotic disorder or severe sensory disabilities are excluded. Patients who have drug or alcohol related abuse or dependence as main diagnosis are also excluded. These exclusion criteria are checked in patient files.

### Recruitment

The PCP screens for eligible patients by searching the database of the primary care practice using the following indicators [33]: antidepressants prescription, strong free text indication of depression or a history of depression according to the International Classification of Primary Care (ICPC) codes in the PCP’s patient files. Subsequently, the PCP approaches potentially eligible patients with global information about the study, contact information and a global screening form comprising three questions: 1) did you experience 2 or more depressive episodes? 2) are you currently recovered? and 3) did the last episode end longer than 8 weeks- and no longer than 2 years ago?. If a patient is eligible for the trial based on the global screening and if the patient agrees upon receiving more information, he is sent an extensive study information letter and an informed consent form for participation in the trial. Consenting patients are assessed for their eligibility in more detail using the SCID-I. Eligible patients who sign informed consent enter the trial.

### The intervention

#### Self-help

The intervention consists of nurse-led self-help treatment based on PCT [21,34]. Patients are offered a self-help book including background literature for further reading and assignments. This self-help book enables patients to follow the treatment in the privacy of their own homes and at a pace that suits them best. The treatment consists of eight weekly modules with a fixed structure and it takes approximately 1.5 hour each week to complete the assignments. Additionally, patients fill out the electronic Q-IDS-SR weekly to monitor the severity of their depressive symptoms.

#### Nurse-led support

The nurse lends minimal support to help patients work through the self-help intervention. Prior to the start of the treatment, a face-to-face meeting with the nurse (with a maximum duration of 30 minutes) is planned at the primary care practice. This meeting involves a discussion of current symptoms, motivational interviewing, psycho-education on the course and treatment of recurrent depression and an introduction to the nurse-led self-help treatment on the basis of the self-help book. Afterwards, the self-help treatment starts and eight weekly telephone contacts (at a maximum of 15 minutes) follow, initiated by the nurse. During these telephone contacts the nurse explores how the patient fares with the self-help treatment according to a strict protocol. In the weekly contacts the nurse asks the following questions: 1) did the patient fill out the electronic Q-IDS-SR questionnaire? 2) did the patient read and understand the literature belonging to that week? 3) did the patient complete the accompanying assignments? and 4) what difficulties did the patient experience in his assignments? After answering these questions, patients are shortly introduced to next week’s literature and exercises. The contact is supportive, activating and facilitating and the nurse does not engage in a therapeutic relationship with the patient. If a nurse notices the emergence of depressive symptoms during a regular phone-contact or a patient brings up feeling depressed, the nurse emphasizes specific parts of the treatment that may help the patient to better cope with these symptoms in order to prevent recurrence. If a patient expresses suicidal thoughts the PCP is notified immediately. After each contact the nurse summarizes the conversation (including the questions) in an electronic journal using a checklist. This journal is a way to both monitor and promote treatment integrity on the side of the patient (did the patient read and apply the literature) and the nurse (did the nurse go through all the questions).

### Training and supervision of nurses

The mental health nurse attends an one-day training, during which attention is paid to the protocol and content of PCT and to guiding a self-help treatment. The nurse is also taught to let recovered patients deal with symptoms. Since participating nurses already have experience with cognitive therapy, an one-day training is sufficient. The training is delivered by two trained psychologist. To detect adherence and/or competence issues, audiotaped telephone contacts with two patients are evaluated during supervision sessions for each nurse, before the actual start of the trial. During the trial, nurses can contact their supervisors at any time for additional questions and feedback.

### Usual care

In both treatment conditions, treatment as usual involves usual care (i.e. standard/routine treatment, including no treatment) as typically provided by the PCP according to the Dutch PCP clinical guidelines (NHG-guidelines) [35]. These guidelines recommend continuation of treatment with AD, preventive psychological treatment or both, depending on the distress, level of dysfunctioning, psychological or physical co-morbidity and preferences of the patient.

In this trial, usual care is not restricted during the period from entry to end of follow-up. By adding no restrictions to usual care, the findings of this study are more generalisable. There usually is some inter-practice variation in treatment despite the clinical guidelines. It is therefore important to obtain a clear understanding of what (additional) treatments are received by patients in both arms of the trial. To that end, data is collected on health care utilization, using the TiC-P [36] – the most commonly used health care receipt interview in the Netherlands (see below for more details on the measurements) and the Medication Adherence Questionnaire (MAQ) [37]. As the use of usual care in both arms in this trial brings the risk of behavioural change by caregivers and patients because of the information that is supplied [38], minimum information is supplied to any participating person (see Discussion for further details).

### Sample size

To calculate the sample size of the intervention and control group, we combined rudimentary findings from previous randomised controlled trials (RCT’s), which resulted in a mean recurrence rate of 33 % after 2 years of follow-up versus 67 % in the control group (active and non-active control). Based on this we assume a risk reduction of 20 % rate in this study between the two conditions. To detect this 20 % risk reduction in a 2-sided test at alpha = 0.05 and a power of 1-beta = 0.80, 107 patients in each condition are required. Compensating for loss to follow-up of 20 % over the whole 15 months follow-up, requires (107/0.8=) 134 participants at baseline in each trial arm.

### Randomisation

Patients who are eligible for the trial and who have given their informed consent, are randomised to ‘nurse-led self-help treatment plus usual care’ or ‘usual care alone’. An independent researcher performs randomisation centrally (Random Allocation Software version 1.0.0), using a blocked randomisation scheme with blocks of 2 patients. The researchers receive the participant’s number and automatically random generated condition in the trial by email. Randomisation is pre-stratified for the number of previous depressive episodes (2 or 3 episodes versus 4 or more episodes). The rationale for stratification based on this variable is that several subgroup analyses suggest that PCT is more effective in ‘high risk’ patients, meaning patients with a history of 4 or more episodes on a lifetime basis [21,39,40].

### Outcome measurements

For an overview of assessments at baseline, during the intervention and during follow-up, see Table 1. The primary researcher (KB) conducts collection and analysis of the data with help of a research assistant (SK).

**Table 1:** overview of assessments

**Measure**	**Description**	**T0**	**w1,8**	**T1**	**T2**	**T3**	**T4**	**T5**
***Interviews***								
SCID-I [32]	DSM-IV Axis I Disorders	+		+	+	+	+	+
***Self-report measures***								
Q-IDS-SR [41]	Depressive symptoms	+	+	+	+	+	+	+
EQ-5D [42]	Quality of life	+		+	+	+	+	+
SF-12 [43]	Quality of life	+		+	+	+	+	+
TIC-P [36]	Direct/indirect costs	+		+	+	+	+	+
4DSQ [44]	Comorbid psychopathology	+				+		+
General self- efficacy scale [45]	Self-efficacy	+				+		+
FSS[46]	Severity of fatigue	+						
MPQ-DLV[47]	Severity/evaluation of pain	+						
MAQ [37]	Medication Adherence	+		+	+	+	+	+
CSQ-8 [48]	Satisfaction							+

T0 = baseline, w1,8 = week 1–8, T1 = 3 months, T2 = 6 months, T3 =9 months, T4 = 12 months, T5 =15 months.

#### Demographics

At baseline the socio-demographic characteristics of participants are collected (age, gender, educational level, marital status, etc.). The number and duration of previous depressive episodes, age of onset of first depressive episode and kind of treatments received are also assessed at baseline.

#### Primary outcome

The primary clinical outcome is the cumulative recurrence rate of depression meeting DSM-IV criteria for a major depressive episode [49]. Recurrence of depression (current or since the last assessment point) is assessed in both arms with the SCID-I [32] at 3, 6, 9, 12 and 15 months follow-up by a trained researcher and research assistant. Interviews are randomly audiotaped and evaluated for integrity reasons. The incidence rate in the intervention group is compared to the incidence rate in the control group and thus expressed as the (cumulative) incidence rate ratio.

#### Secondary outcomes

Secondary clinical outcomes include health related quality of life (measured with both the ‘EuroQol’ (EQ-5D)[42] and ‘Short Form-12’ (SF-12)[43]), severity of depressive symptoms (measured with the Q-IDS-SR [41]), co-morbid distress, anxiety and somatisation (measured with the ‘Four Dimension Symptom Questionnaire’, 4-DSQ [44]) and self-efficacy (measured with the ‘General Self Efficacy Scale’, GSES [45]). Quality adjusted life years (QALY’s) are calculated based on both the EuroQol [42] and SF-12 [43], using the Dutch tariff estimated by Lamers et al. [50] and using Brazier’s algorithm [51], respectively. All secondary clinical outcomes are measured at baseline and at 9 and 15 months follow-up.

#### Putative effect-moderators

Several risk factors for recurrent depression have been identified [52] and may also be relevant for predicting treatment response. These risk factors include non-adherence, demographic factors such as age and gender, high number and longer duration of previous episodes, younger age at the onset of the first depressive episode [53], presence of residual symptoms [54], low socioeconomic status [55], low self-efficacy for managing depression [55] and also symptoms of pain [56] and fatigue [57] appear to be risk indicators for imminent recurrence of depression. The type of treatment received during previous depressive episodes (psychological intervention/AD/no care) may also be relevant for treatment response. Patients who already had a psychological intervention may have the benefit of possible long-term protection for recurrence [58]. All of these factors are therefore assessed as putative effect-moderators.

#### Cost measures

Cost-effectiveness is evaluated from a societal perspective meaning that the costs of the intervention, other health care utilization costs, patients' out-of-pocket costs and costs due to productivity losses are included in the economic evaluation. Health care utilization is measured using the TiC-P [36] at baseline and 3, 6, 9, 12 and 15 months follow-up. Medical costs that are assessed include costs related to the intervention, medication use, hospital admissions, and contacts with other healthcare professionals. For the valuation of health care utilization, standard prices published in the Dutch costing guidelines are used [59]. Medication use is valued using prices of the Royal Dutch Society for Pharmacy, including the costs of prescription by the PCP and the pharmacist’s dispensing costs. A cost price for the nurse-led self-help intervention is calculated using a bottom-up approach and will account for costs for personnel, patient materials and rental of practice spaces. Costs and effects exceeding 12 months follow-up are discounted, in accordance with the Dutch guideline for economic evaluation in health care [60]. Costs of productivity losses are estimated using the friction cost method [61]. In a secondary analysis, the human capital method is used to estimate productivity losses.

### Analyses of clinical outcomes

Standard descriptive methods (e.g. frequencies, percentages and means) are used to summarize the demographic and clinical features of the intervention and control group and to check whether the randomisation has resulted in a well-balanced design.

Analyses are conducted according to the intention-to-treat principle, meaning that all patients who have been randomised are included in the analyses. Missing endpoints are imputed using the Expectation-Maximization (EM) algorithm. To gauge the robustness of the outcomes, this analysis is repeated while using a Multiple-Imputation (MI) approach. All tests are conducted at P <0.05, 2-tailed. Additional completer analyses for all patients that attended at least 80 % of the telephone sessions are performed.

#### Analysis of primary outcome

Cumulative recurrence rate ratios are estimated using a Poisson regression model. Number-needed-to-be-treated (NNT) is calculated as the inverse of the risk difference (RD) which is estimated using a linear probability model. Data-analysis takes into account that data are clustered within primary care practices and patients. The nested data structure entails violation of the usual assumption that data are uncorrelated. Therefore, all analyses are design-based, taking the clustered data structure into account, using Stata’s [62] procedures for clustered data [63].

#### Analysis of secondary outcomes

The effect of the self-help treatment on health related quality of life, symptom severity, co-morbid distress, anxiety and/or somatisation and self-efficacy is analysed by regressing these secondary endpoints with the randomisation condition, while correcting for baseline values.

#### Analysis of effect-moderation

Moderator analyses are conducted for socio-demographic and clinical variables. Subgroups that show particularly good response to the intervention are identified by regressing (*P* < 0.05) depression severity (measured with Q-IDS-SR) on the interaction term of treatment and baseline characteristics of the patients, namely number of previous depressive episodes, previous and current treatment, age of onset of first depression, self-efficacy in managing depression, symptoms of pain and fatigue and socio-demographic characteristics like gender, marital status, age and socio-economic status.

### Analysis of economic data

Missing cost- and effect data are imputed using multiple imputation according to the MICE algorithm developed by van Buuren [64]. Costs typically have a highly skewed distribution. Policy makers want to have information on the difference in mean total costs between the two treatment groups in order to estimate the total health care budget needed for a specific condition [65]. Therefore, bias-corrected and accelerated bootstrapping with 5000 replications is used to estimate 95 % confidence intervals around the mean difference in total costs between the treatment groups.

Both a cost-effectiveness analysis (CEA, with depression-free person years as the clinical end term) and as a cost-utility analysis (CUA, with incremental costs per quality adjusted life years (QALY) gained as the clinical end-term) are performed. Bootstrapping is used to estimate the uncertainty surrounding the incremental cost-effectiveness ratios (ICERs) which are graphically presented on cost-effectiveness planes. Cost-effectiveness acceptability curves and net monetary benefits are also estimated [66].

An incremental net benefit regression (INBR) analysis is conducted to address the research question in which groups the intervention is likely to be particularly cost-effective, analogous to the moderator analyses for clinical endpoints. The same set of variables is used in these INBR analyses. The incremental net benefit is calculated as Eλ – C. The first term is the number of units of effectiveness gained in the intervention group in comparison with the control group multiplied by the amount (λ) society is willing to pay (WTP) for a unit of effect gained. Because λ is unknown, we use a likely WTP-range. The product term is subtracted by the difference in costs between the groups yielding the net benefit expressed in monetary terms. Incremental net-benefits are analysed using a regression analysis approach [67] and helps to identify sub-groups for which the intervention is particularly cost-effective.

### Patients’ satisfaction

At 15 months patients’ satisfaction is assessed using the Client Satisfaction Questionnaire (CSQ-8) [48] in both the intervention and control group. Additionally, 20 ‘experimental’ patients who responded best, and 20 ‘experimental’ patients who responded worst in terms of recurrences are approached for an in-depth (qualitative) interview. Comparing responses from both groups may help to increase understanding what aspects of the intervention must change or remain intact.

## Discussion

Given its recurrent character, new minimal interventions are needed to prevent new episodes of MDD. As patients with recurrent MDD account for great societal costs, an intervention to prevent recurrence in the maintenance phase will potentially lead to great reduction in health care utilization and costs of absenteeism and presenteeism. This is as far as we are aware of, the first study that examines the cost-effectiveness of a nurse-led, cognitive treatment based self-help for patients with recurrent MDD in primary care. This innovative self-help format ensures that patients can complete the treatment in their own time and at their own homes, which makes it easy accessible. Besides, being led by nurses, the treatment is expected to be economically affordable and sustainable.

The effect of several socio-demographic and clinical variables on treatment response is assessed. This might lead to insights that will lead to the development of more targeted interventions.

The RCT-design of this trial is considered the ultimate test of a medical hypothesis, and is the support of evidence-based medicine. By adding no restrictions to usual care in this RCT, the findings of this study will be more generalisable.

Risks for adverse events in patients in the intervention arm are very low due to the psychological character of the intervention and because there is no restriction to usual care. In the case of a patient expressing suicidal thoughts, the PCP is notified. These procedures are made explicit in the informed consent papers and protocols.

A limitation of this trial is that there might not be a big contrast in primary outcome (cumulative rate of recurrence) between the 2 arms of the trial at the end of follow-up, because both arms include usual care. Another limitation is, as in any trial that involves psychological treatment, that it is not possible, due to the design of the intervention, to blind patients, health care providers and researchers to the patient’s randomised condition. Therefore it is not possible to prevent any behavioural change during the course of the trial. Patients’ behaviour in the control arm might be influenced by reading the study information letter and a PCP’s choice regarding usual care might be influenced by the knowledge of a patient starting the intervention. Nurses might be influenced in their support for patients when guiding the self-help treatment because of their knowledge of the patients’ additional care. The information given to PCP’s, nurses and patients in both arms is therefore limited to a minimum to overcome this limitation.

## Competing interest

The authors declare that they have no competing interests.

## Authors’ contributions

KB and SK drafted this paper which was added to and modified by HvdH, AvS, JB, FS, CB, and HvM. CB modified the content of PCT to the content of the nurse-led self-help treatment. KB, CB, HvdH, AvS, FS and HvM contributed to the design of the study and JB, FS and KB contributed to the analytic strategy. All authors read and approved the final manuscript.

## Ethical principles

The study protocol was approved by the Ethics Committee of the VU University Medical Centre and will be conducted according to the principles of the Declaration of Helsinki (version 2004) and the Medical Research Involving Human Subjects Act (WMO).

## Pre-publication history

The pre-publication history for this paper can be accessed here:

http://www.biomedcentral.com/1471-244X/12/59/prepub
